# AMPK Activity: A Primary Target for Diabetes Prevention with Therapeutic Phytochemicals

**DOI:** 10.3390/nu13114050

**Published:** 2021-11-12

**Authors:** Min-Yu Chung, Hyo-Kyoung Choi, Jin-Taek Hwang

**Affiliations:** 1 Personalized Diet Research Group, Korea Food Research Institute, Jeonju 55365, Korea; mic07002@kfri.re.kr (M.-Y.C.); chkyoung@kfri.re.kr (H.-K.C.); 2 Department of Food Biotechnology, University of Science and Technology, Daejeon 34113, Korea

**Keywords:** diabetes, AMP-activated protein kinase, medicinal plants, ingredient

## Abstract

Diabetes is a metabolic syndrome characterized by inadequate blood glucose control and is associated with reduced quality of life and various complications, significantly shortening life expectancy. Natural phytochemicals found in plants have been traditionally used as medicines for the prevention of chronic diseases including diabetes in East Asia since ancient times. Many of these phytochemicals have been characterized as having few side effects, and scientific research into the mechanisms of action responsible has accumulated mounting evidence for their efficacy. These compounds, which may help to prevent metabolic syndrome disorders including diabetes, act through relevant intracellular signaling pathways. In this review, we examine the anti-diabetic efficacy of several compounds and extracts derived from medicinal plants, with a focus on AMP-activated protein kinase (AMPK) activity.

## 1. Introduction

The incidence of metabolic syndrome disorders which include diabetes, high blood pressure, and fatty liver, increases significantly with age in the world [1]. Globally, there were an estimated 463 million people living with diabetes as of 2019. Diabetes is a metabolic syndrome characterized by inadequate blood glucose control, accompanied by frequent urination, increased thirst, and increased appetite [2,3]. When diabetes becomes severe, complications such as cardiovascular disease, stroke, chronic kidney disease, and foot ulcers can arise [4]. While type 2 diabetes is caused by insulin resistance, in which insulin is secreted properly but the cells do not respond appropriately [5,6], type 1 diabetes is more associated with abnormal insulin production. Recently, strategies to prevent diabetes with dietary supplements together with dietary control have been recommended [7,8]. As such, dietary supplements that may act to prevent metabolic syndrome have been developed commercially, and further efforts are being made to identify the active substances responsible for the effects [7,8,9].

It has been reported that exercise activates a specific intracellular signaling system that has positive effects on various aspects of metabolic syndrome including diabetes [10]. Two signaling pathways are widely implicated in diabetes [10,11]. One consists of insulin being secreted from pancreatic cells and binding to the insulin receptor of the target cell due to increases in blood glucose concentrations, after which the insulin receptor is activated and insulin receptor substrate (IRS)-1 activity is increased. This activates the downstream phosphoinositide (PI)3-kinase and Akt signaling cascades, which in turn promotes the translocation of the glucose transporter to the cell wall and increases glucose uptake, resulting in an improvement in diabetic symptoms [10,11]. Interestingly, exercise can increase glucose uptake independent of PI3 kinase activity [10,11,12]. The second pathway involves an insulin-independent signaling system, which is thought to have greater translational relevance for diabetes management [13,14]. When muscles repeat contractions and relaxation during exercise, improvements in insulin sensitivity and glucose tolerance are stimulated [15,16]. Further analysis of these signaling pathways has shown they increase mitochondrial biogenesis and promote the expression of glucose transporter type (Glut)4 [15,16,17]. Muscle movement also leads to an increase in intracellular Ca^2+^ and the increased calcium concentrations activate calcium/calmodulin-dependent protein kinases (CaMKs) [17,18]. The CaMK family consists of CaMK I, II, and IV isoforms [17,18]. It has been reported that increased calcium from exercise activates CaMKs while also increasing the activity of markers that enhance glucose uptake [18,19]. The peroxisome proliferator-activated receptor gamma coactivator (PGC)-1α plays a central role in energy metabolism as a transcriptional coactivator of fat metabolism and carbohydrate metabolism and also plays an important role in mitochondrial biogenesis induced by exercise [20]. PGC-1α promotes muscle remodeling to a fiber type composition, resulting in a lower glycolytic metabolism [21]. PGC-1α is a co-activator of a variety of transcription factors, including thyroid hormone receptors, glucocorticoid receptors, estrogen receptors, estrogen-related receptors, and non-nuclear receptor transcription factors such as the myocyte enhancer factor (MEF)-2 and the family of forkhead O-box (FOXO) transcriptional factors [21,22,23].

CaMKK has been reported to play a role in the AMP-activated protein kinase (AMPK) activation responsible for contractions in skeletal muscle [18,24]. Transcription factors are regulated by the AMPK protein, which is a key player in metabolic regulation, and transcription factors are regulated by PGC-1α overlap in many roles, hence suggesting that PGC-1α might be a central mediator of AMPK-induced gene expression [20,25]. In support of this hypothesis, several studies have suggested that AMPK activity increases the expression of PGC-1α and AMPK activity is also required for PGC-1α activity to regulate mitochondrial and glucose metabolism [20,25,26]. A more potent interaction between AMPK and PGC-1α has recently been reported where AMPK can directly bind to and phosphorylate PGC-1α, thereby increasing the transcriptional activity of PGC-1α [26,27]. However, the precise mechanism of action and how it binds and phosphorylates the target remains unclear. Nevertheless, the phosphorylation of PGC-1α by AMPK is needed for the detection of the energy state following exercise and the regulation of the transcriptional programs that control glucose metabolism. For this reason, AMPK activity is often a key mediator of the effects of ingredients derived from medicinal plants in the study of diabetes prevention.

## 2. AMPK-Mediated Signaling in Diabetes

AMPK is a serine/threonine kinase that functions as a cellular energy-sensing protein that is activated by reducing glucose availability [28] or adenosine triphosphate (ATP) in cells [29]. The kinase is a heterotrimer composed of three subunits, the α-subunit, β-subunit and γ-subunit [29,30]. The α-subunit is in turn composed of α1 and α2 isoforms, while the β-subunit is composed of β1 and β2 isoforms, and the γ-subunit includes the γ1, γ2, and γ3 isoforms in humans [29,30]. The AMPK α-subunit is a catalytic factor that increases AMPK activity when phosphorylation of thr172 by an upstream kinase occurs [29,30]. The β-subunit plays an important role in forming the functional α–β–γ complex, and the central region of the β-subunit harbors a glycogen-binding domain (GBD) [30,31]. The γ-subunit contains cystathionine beta synthase (CBS) domains that are important for sensing the AMP:ATP ratio, a critical factor for AMPK activation [32]. AMPK is activated under conditions that increase the ratio of adenosine monophosphate (AMP) to adenosine triphosphate (ATP), such as exercise and metabolic stress [30,31,32]. Studies on the effects of exercise, hypoxia, and ischemia have shown that when the AMP:ATP ratio increases, AMPK is activated by AMPK kinase and binds to AMP to induce a conformational change that blocks the ATP consumption pathway and activates the ATP generation pathway [30,31,32]. Once activated, AMPK suppresses lipid and sterol synthesis by inhibiting the activity of acetyl-CoA carboxylase, leading to reduced glycogen storage by glycogen synthases. Conversely, the catabolic pathway is promoted in order to prevent ATP depletion [30,31,32]. AMPK phosphorylates proteins are involved in the translocation of the glucose transporter and promote the translocation of Glut4 to the cell membrane, thereby increasing glucose uptake [16]. AMPK also activates lipases and promotes the release of fatty acids, thereby promoting β-oxidation [16]. Beta-oxidation and blood glucose absorption are representative pathways responsible for generating energy [16]. This central role means that AMPK can influence the progression of various diseases. In the case of obesity, the consumption of energy is required to accumulate fat, and this pathway can be blocked by AMPK activity [33]. As such, numerous natural anti-obesity products and medicines target AMPK activation [8,33,34,35]. In terms of anti-obesity mechanisms, activated AMPK phosphorylates and inhibits ACC, which consequently decreases malonyl-CoA, inhibiting fatty acid synthesis and simultaneously increasing mitochondrial β-oxidation [33,34,35]. In the case of diabetes, glucose absorption eventually triggers the process of energy generation in cells, so when AMPK is activated, glucose absorption is enhanced and a pathway that creates energy in target cells is activated [30,34]. Recently, a new study reported that AMPK is activated by an interaction with long chain fatty acyl-CoA (LCFA-CoA) esters which play a critical role in cellular energy metabolism as energy sources and signaling molecules [36,37]. The study demonstrates that LCFA-CoA esters allosterically activate AMPK β1-containing isoforms to upregulate fatty acid oxidation through the phosphorylation of acetyl-CoA carboxylase [37]. LCFA-CoA esters accelerate their own oxidation by acting as allosteric inhibitors of acetyl-CoA carboxylase, and in turn reduce the malonyl-CoA production [38], inducing an increase in mitochondrial β-oxidation. However, research related to this is at a very early stage, and more studies need to be conducted for an overall discussion. Ultimately, the activity of AMPK plays a role in improving diabetic outcomes.

There has been a study about the role of AMPK in controlling nitric oxide (NO)-induced beta cell death. AMPK promotes the functional recovery of β-cell oxidative metabolism, and mitigates the activation of pathways mediating cell death, including caspase-3 following NO exposure [39]. In addition, little is known about how AMPK activation affects insulin secretion. A study conducted by Bai et al. demonstrated that berberine inhibits glucose-stimulated insulin secretion from rat islets, which is attributed to the decreased oxygen consumption rate and ATP production independent of AMPK activation [40]. This study concludes that the exact mechanism remains to be further explored [40]. Based on these previous studies, this review aims to emphasize AMPK-mediated signaling in insulin resistance and sensitivity (type 2 diabetes), and the preventive roles of foods or foods contained phytochemicals.

## 3. Pharmacological AMPK Activator

AMPK is a serine/threonine kinase that functions as a cellular energy-sensing protein that is activated by adenosine triphosphate (ATP) depletion in cells [29]. The kinase is a heterotrimer composed of three subunits, the α-subunit, β-subunit, and γ-subunit [29,30]. The α-subunit is in turn composed of α1 and α2 isoforms, while the β-subunit is composed of β1 and β2 isoforms, and the γ-subunit includes the γ1, γ2, and γ3 isoforms in humans [29,30]. The AMPK α-subunit is a catalytic factor that increases AMPK activity when phosphorylation of thr172 by an upstream kinase occurs [29,30]. The β-subunit plays an important role in AICAR (5-aminoimidazole-4-carboxamide riboside) which is an endogenous natural metabolic intermediate derived from purine biosynthesis in all organisms [41]. It has also been reported that AICAR reduces the expression of genes such as PEPCK (phosphoenolpyruvate carboxykinase) and G6Pase (glucose-6-phosphatase), which are important for gluconeogenesis, thereby inhibiting glucose production [42]. In addition, AICAR increases blood glucose absorption, increases the expression of Glut4, and promotes AS160 phosphorylation in muscle tissue. It has also been reported that AMPK may play an important role in the pathogenesis of type 2 diabetes and toxicity in pancreatic β-cells [43]. AICAR treatment of pancreatic β-cells can increase fatty acid oxidation, improve pancreatic β-cell function, and reduce apoptosis caused by hyperglycemia [44,45].

Metformin (1,1-dimethylbiguanide hydrochloride), one of the most common treatments for diabetes, is derived from guanidine and reduces blood glucose levels via AMPK activation [46]. Recently, a mechanism for reducing blood glucose by decreasing bile acid absorption and increasing GLP-1 secretion has also been reported [47]. Although metformin acts as a first-line treatment approach against type 2 diabetes, it often causes gastrointestinal side effects, including nausea, vomiting, and diarrhea [48].

Thiazolidinediones (TZDs), also known as glitazones, represent another pharmacological treatment option for diabetic patients. TZDs activate peroxisome the proliferator-activated receptor (PPAR)γ primarily in adipose tissue, mitigating insulin resistance. However, TZD also increases the risk of heart failure, bone fractures, weight gain, fluid retention, and edema [49]. A recent study has demonstrated that the TZD pioglitazone activates AMPK independent of PPARγ [50]. Pioglitazone selectively protects beta cells against high glucose toxicity via AMPK activation, regulating the TRAP1/HSP75–Glutaminase 1 (GLS1) interaction, leading to an increased GSH/GSSG ratio as well as inhibiting mTORC1-mediated maladaptive ER stress in beta cells [50].

A-769662 is a direct allosteric activator of AMPK [51]. Findings from an in vivo study using *ob*/*ob* mice suggest that the administration of A-769662 decreases plasma glucose and triglyceride levels, reducing hepatic triglycedride and the expression of phosphoenolpyruvate carboxykinase (PEPCK), glucose-6-phosphatase (G6Phase), and fatty acid synthase (FAS), as well as reducing weight gain [52]. It has also been demonstrated that A-769662 directly stimulates partially purified liver AMPK and inhibits fatty acid synthesis in rat primary hepatocytes. In addition, treatment with A-769662 (for short-term reductions in hepatic malonyl CoA levels and VCO2/VO2 respiratory exchange ratio) increased whole body fatty acid oxidation in normal Sprague Dawley rats [52]. Such effects of A-769662 are associated with its role as an AMPK activator. Strembitska et al. demonstrated that A-769662 inhibited insulin-stimulated Akt phosphorylation and nitric oxide (NO) synthesis in human macrovascular extracellular components (EC) from umbilical veins (HUVECs) and aorta (HAECs). These A-769662-mediated actions were not attenuated when AMPK activity was inhibited with SBI-0206965 and were not affected by the AMPK activating compound 991 or AICAR treatment. These findings together suggest that the inhibitory action of A-769662 in EC is independent of AMPK [53].

## 4. Natural Plant Phytochemicals That Activate AMPK

A previous study has demonstrated that the areca nut extract, which is enriched with catechin-based procyanidins, elicits anti-inflammatory effects in vitro and in vivo [54]. Areca nut procyanidin (ANP) inhibits gluconeogenesis by reducing PEPCK and G6Pase [54]. In addition, administration of ANP reduced fasting blood glucose levels, as well as PEPCK and G6Pase activity in MLD-STZ mice. Interestingly, the levels of AMPK expression and phosphorylation were restored when compared to the ANP-treated MLD-STZ-mice [54]. These findings indicate that ANP is effective in regulating blood glucose via gluconeogenesis-related enzymes and AMPK.

In a mouse model of type 2 diabetes, dieckol, a phlorotannin isolated from *Ecklonia cava* was found to significantly reduce blood glucose levels, serum insulin, and body weight [55]. This phenomenon was observed to increase the activity of antioxidant enzymes superoxide dismutase (SOD), catalase (CAT), and glutathione peroxidase (GSH-px) in liver tissue, as well as increase the phosphorylation levels of AMPK and Akt in muscle tissue [55]. This study suggests that dieckol can be used as a treatment for diabetes due to its role in AMPK activation.

Hsu et al. evaluated the anti-diabetic efficacy of pterosin A, a natural product isolated from several fern plants [56]. Administration of pterosin A for 4 weeks dramatically improved hyperglycemia and glucose intolerance in a mouse model without any observed side effects. These phenomena were accompanied by significantly increased phosphorylation of AMPK and Akt in the muscle [56]. In addition, pterosin A increased glucose uptake and AMPK phosphorylation in cultured muscle cells [56]. These results suggest that the anti-diabetic effect of pterosinA is achieved through the activation of AMPK.

Saffron is a species of flowering plant that exhibits various physiologically active properties, including anti-cancer and anti-inflammatory effects. Kang et al., investigated the glucose metabolism-related signaling pathways in skeletal muscle cells associated with the glycemic control effects elicited by saffron [57]. Saffron significantly increased the phosphorylation of AMPK, acetyl-CoA carboxylase (ACC), and mitogen-activated protein kinase (MAPK) to increase glucose uptake, and it was confirmed that this phenomenon occurred independently of the PI3 kinase/Akt pathway [57].

Berberine is a component derived from natural products and is present in various plants including goldenseal, oregon grape, and tree turmeric. According to the results of a study on the anti-diabetic effects of berberine, it was confirmed that the compound improved diabetic symptoms via AMPK activity in the liver and muscle [58]. In addition, it was confirmed that berberine acted via AMPK regulation of the insulin gene promoter in mice, and it was found that insulin resistance and glucose tolerance were improved [58]. In another study, the anti-diabetic effects of berberine were accompanied by a reduced expression of PEPCK and G6Pase, which are genes involved in gluconeogenesis in the liver [59]. Berberine also reduces hepatic steatosis by regulating fatty acid synthase (FAS), the forkhead transcription factor O1 (FoxO1), the sterol regulatory element-binding protein 1c (SREBP1), and the carbohydrate responsive element-binding protein (ChREBP) [59]. In addition, berberine plus sodium caprate increased the bioavailability of berberine without specific mucosal damage, and it was shown that hepatic gluconeogenesis can be inhibited via AMPK activity [60].

The effect of processed Aloe (Aloe QDM) on metabolic syndrome was also evaluated by Shin et al. Supplementation with Aloe QDM lowered body weight, fasting blood glucose levels, plasma insulin, and leptin levels and increased adiponectin, thereby improving glucose tolerance in a high-fat diet mouse model [61]. Aloe QDM also increased plasma adiponectin levels and insulin sensitivity through AMPK activity.

*Larix laricina K. Koch* has been traditionally used as a medicinal plant in Canada. However, there were previously no scientific studies that had investigated its anti-diabetic and anti-obesity effects. Researchers then evaluated the anti-diabetic and anti-obesity effects of *Larix laricina* using a mouse model [62]. Administration of a high-fat diet plus *Larix laricina* lowered glycemia levels, improved insulin resistance, and reduced abdominal fat and body weight. This increased energy expenditure and improved mitochondrial function and ATP synthesis [62]. In vitro results also showed that *Larix laricina* increased glucose uptake and inhibited fat differentiation via activation of AMPK [63].

Resveratrol, a natural substance derived from grapes, is well known for its various physiologically-active effects and is consumed as a supplement to promote good health [64]. Numerous studies have reported on the preventive effects of resveratrol on metabolic syndrome [64,65]. It was found that the supplementation of resveratrol in a high-fat diet animal model or *db*/*db* mouse reduced blood glucose levels and triglycerides through the increase in and control of various downstream targets. In addition, high concentrations of resveratrol were shown to increase hepatic glycolytic gene expression and enzyme activity. From this, it can be seen that AMPK activity is important for resveratrol to improve metabolic syndrome via its regulation of various targets. Piceatannol, a metabolite of resveratrol, is also a well-known AMPK activator [66]. Researchers evaluated the anti-diabetic effect of piceatannol in cell and animal models, showing that it increases glucose uptake in L6 muscle cells, activates AMPK, and promotes glut4 translocation. Piceatannol also reduced blood glucose and improved impaired glucose tolerance in diabetic animal models [66]. Taken together, these findings suggest that the activity of AMPK is important in the anti-diabetic effects of piceatannol.

Baicalein is a flavone abundantly present in the roots of *Scutellaria baicalensis* and *Scutellaria lateriflora*. In a recent study, the effect of baicalein on metabolic syndrome was evaluated in a high-fat diet mouse model, demonstrating its effects on metabolic syndrome by improving various biomarkers [67]. Baicalein improved insulin sensitivity and inflammatory markers via the inhibition of the MAPK signaling pathway and the activation of the IRS1/PI3K/Akt signaling pathway. The lipid-lowering effect was elicited by suppression of SREBP-1c and PPARγ activity and increasing fatty acid oxidation [67]. Playing a central role in these effects was the signaling activity mediated by AMPK.

Insulin resistance is associated with several forms of inflammation, with inflammatory cytokines playing an important role [68]. Studies have sought to investigate the effects of *Momordica charantia*, also known as bitter melon, on inflammation and insulin resistance. Triterpene 5β,19-epoxy-25-methoxy-cucurbita-6,23-diene-3β,19 purified from *M. charantia L.-diol* (EMCD) exhibited hypoglycemic effects in FL83B cells [68]. TNF-α-induced inflammation was inhibited by EMCD, which in turn inhibited the inflammatory markers inducible nitric oxide synthase (iNOS), the p65 subunit of nuclear factor-κB (NF-κB), protein-tyrosine phosphatase-1B, TNF-α, and interleukin. It was also shown to inhibit the expression of interleukin (IL)-1β, while EMCD induced AMPK expression [68]. Another study reported that bitter melon triterpenoids increased glut4 translocation accompanied by the activation of AMPK, and consequently counteracted insulin resistance [69]. Following evaluation in a cell model to clarify this mechanism, CaMKKβ was identified as an upstream kinase responsible for the triterpenoid-induced AMPK activation.

In our recent study, we evaluated the anti-diabetic effects and mechanisms of action responsible for the various physiological activities of tangeretin (5, 6, 7, 8, 4′-pentamethoxyflavone), which is abundantly present in citrus fruits. Tangeritin was confirmed to increase AMPK activity as well as phosphorylation of AS160 [70]. This phenomenon was confirmed in a high-fat diet mouse model, accompanied by effects on several aspects of metabolic syndrome including weight gain, glucose tolerance, and total cholesterol, while also suppressing the secretion of various adipokines. Interestingly, the activity of AMPK in rat muscle tissue was also confirmed to be increased in the tangeretin-fed group, suggesting that the anti-diabetic effects of tangeretin require AMPK activity.

According to the results of an evaluation of the anti-diabetic effects of a phenolic compound isolated from *Ishige foliacea*, it was reported that octaphlorethol A increased glucose uptake, promoted glut4 translocation to the plasma membrane, and increased Akt and AMPK activity in a cell model [71].

Chlorogenic acid is a phenolic compound found in coffee and various fruits and vegetables that is known to have beneficial effects on health [72,73]. Research has shown that chlorogenic acids increase glucose transport in soleus muscle and L6 myotubes. This phenomenon can be abrogated by AMPK inhibitor compound C and AMPKa1/2 siRNA knockdown [73], demonstrating that chlorogenic acid-induced glucose transport is achieved through the activity of AMPK.

Ginsenoside Rg1, a protopanaxatriol saponin present in Panax ginseng has been reported to elicit various physiological activities, and has been used as a traditional medicine, particularly in Asia, for centuries [74]. A recent study evaluated its effect in type 2 diabetes and revealed that Rg1 increased glucose uptake in chronic insulin-treated muscle cells, concomitant with the activation of AMPK [74], suggesting that activation is required for the improvement of insulin sensitivity in these cells.

Osthole is an important coumarin compound that was discovered in the genus *Cnidium monnieri* (L.) *Cussion*, a traditional Chinese herbal medicine. Researchers have investigated its effects on regulating glucose uptake and related mechanism [75]. Osthole was found to increase the phosphorylation of AMPK and ACC in cell models and increased the translocation of glut4 to the plasma membrane, thereby increasing blood glucose uptake. It was confirmed that the increased AMPK activity by Osthole was achieved by increasing the intracellular AMP:ATP ratio, and the anti-diabetic effects of Osthole were recapitulated in animal experiments [75].

Arctigenin is a lignan found in certain members of the Asteraceae family and has been reported to elicit anti-cancer and anti-viral effects [76]. In L6 myotubes, arctigenin increased AMPK phosphorylation and promoted glucose uptake, while reducing gluconeogenesis and lipid synthesis in primary hepatocytes. In animal studies, arctigenin supplementation reduced gluconeogenesis, lowered blood glucose levels, and improved lipid metabolism [77]. These results demonstrate that arctigenin is an activator of AMPK while improving aspects of metabolic syndrome.

Malva verticillata (MV) seeds contain various polysaccharides and flavonoids that are known to have diuretic effects [78]. The ethanol extract from MV seeds has been shown to increase the phosphorylation of AMPK and its substrate ACC, leading to increased glucose uptake in L6 myotubes [79]. The extract was fractionated with organic solvent to further evaluate potential anti-diabetic effects. The extract fractionated with n-hexane (MVE-H) increased the phosphorylation of AMPK and ACC to the highest levels observed while also improving glucose uptake. In animal experiments, MVE-H reduced blood glucose in *db*/*db* mice and increased the phosphorylation of AMPK and ACC in muscle and liver tissue, reflecting the same results seen in the cell experiments [79].

Natasha et al. demonstrated several anti-diabetic effects of karanjin isolated from the fruits of Pongamia pinnata grown in tropical Asia and Australia in a cell model [80]. Karanjin increased glucose uptake and promoted glut4 translocation to the cell membrane. This effect was accompanied by AMPK activation, while it did not affect the phosphorylation of Akt, another known mediator of anti-diabetic effects [80].

Researchers have also investigated the blood glucose absorption capacity of ReishiMax (RM) containing triterpenes and polysaccharides extracted from the mushroom *Ganoderma lucidum* in a cell model [81]. RM inhibited adipocyte differentiation and reduced various adipogenic markers including peroxisome PPARγ, SREBP-1c, and the CCAAT/enhancer binding protein-α (C/EBP-α). In addition, it was confirmed that RM promoted the phosphorylation of AMPK and increased blood glucose uptake, highlighting the potential anti-obesity and anti-diabetic effects of RM [81].

*Prunus yedoensis Matsum.* is a plant species commonly displayed for ornamental purposes in the United States, Korea, and Japan [82]. Studies have focused on whether the leaf of the plant yields anti-diabetic activity. Prunus yedoensis leaf extract (PLE) was found to increase glucose uptake in L6 myotubes and stimulated the phosphorylation of AMPK and p38 MAPK, and these increases were reversed in the presence of AMPK inhibitor compound C and the p38 inhibitor SB203580 [83]. These findings suggest that AMPK is involved in PLE-mediated increases in glucose uptake.

Circulating glucose levels are balanced by liver glucose production and muscle glucose consumption, and when this balance is disrupted, diabetes can arise [84]. Therefore, excessive hepatic glucose production via gluconeogenesis adversely affects diabetic patients, and the inhibition of gluconeogenesis is a viable therapeutic strategy for diabetes prevention. *Artemisia sacrorum Ledeb.* (Compositae) (PEASL) has been shown to inhibit glucose production in human HepG2 cells, an effect that was restored by treatment with compound C. PEASL was confirmed to stimulate the phosphorylation of AMPK and its downstream target, ACC [85]. Various genes involved in gluconeogenesis such as PGC-1α, PEPCK, and G6Pase were also inhibited by PEASL [85].

The seeds of *Nigella sativa* L. (NS) have been widely used as a traditional medicine, and are known to have anti-inflammatory, anti-diabetic and anti-cancer properties [86]. After inducing diabetes in the rodent Meriones shawi, an ethanol extract from the Nigella sativa seed (NSE) was administered. Under the same conditions metformin (an AMPK activator) was administered, resulting in improvements in blood glucose control and reductions in triglyceride content in liver and muscle tissue. NSE concomitantly stimulated the phosphorylation of ACC (a direct target of AMPK) in the muscle and liver, and increased muscle Glut4 expression [86]. These results demonstrate that the glycemic control associated with improved insulin resistance by NSE treatment is closely linked to the AMPK signaling pathway.

Capsaicin is a type of alkaloid and a bioactive compound present in capsicum that has been widely studied for its beneficial effects on health [87]. Studies have revealed various effects of capsaicin supplementation on metabolic syndrome, including in genetically diabetic mice. Administration of a high-fat diet plus capsaicin in male KKAy mice resulted in reduced levels of fasting blood glucose, insulin, and triglycerides in the plasma and liver, while the expression of inflammatory adipokine genes was also reduced [87]. These effects occurred together with increased AMPK activity in the liver.

Tang-min-ling, a traditional Chinese medicine concoction of various herbs, contains Coptis chinensis Franch as an ingredient [88]. Studies have investigated its effects on insulin resistance in Otsuka Long-Evans Tokushima Fatty (OLETF) rats. Tangminling was shown to be effective in reducing insulin resistance in an oral glucose tolerance test, and decreased serum leptin levels. This was accompanied by increases in AMPK activity and glut4 expression in muscle tissue [88].

The anti-diabetic effects and mechanism of action of Gastrodia elata Blume, traditionally used in East-Asian medicine, have also been investigated. Administration of Gastrodia elata Blume water extract reduced energy intake by enhancing STAT3 phosphorylation and reducing AMPK phosphorylation in the hypothalamus [89].

## 5. Conclusions

In this review, we have summarized reports on various crude extracts and constituents of medicinal plants that are potentially relevant to the prevention of diabetes via AMPK activation (summarized in Table 1). A number of these phytochemicals have shown efficacy in glycemic control, insulin resistance, and glucose tolerance via the activation or inhibition of diabetes-related biomarkers, all of which have been linked to the activity of AMPK. However, despite promising efficacy findings in cell and animal models, further data are needed to characterize the absorption, bioavailability, metabolism, and target exposure of these compounds following ingestion in the human body. In addition, in many cases it remains unclear which precise signaling cascades regulate diabetes-related biomarkers and, in particular, increase the activity of AMPK. Although the traditional use of many of these medicinal plants for their anti-diabetic effects has occurred throughout history, scientific evidence proving their anti-diabetic effects through clinical trials has been lacking. Further studies are needed to provide evidence for the absorption, bioavailability, metabolism, and intracellular absorption of these anti-diabetic medicinal plants in relation to the relevant receptors and signal cascades.

Unfortunately, it is a limitation that this review is unable to capture the effect of these products and AMPK signaling on pathways and processes outside of the muscle and liver. Despite these shortcomings, we are confident that the central role of AMPK in the anti-diabetic efficacy of numerous medicinal plants and active ingredients will continue to be studied, in the hope that new and safe phytochemical-based interventions will be made available to prevent the onset of diabetes.

## Figures and Tables

**Table 1:** Phytochemicals or plants and their target mechanism via AMPK activation in diabetes [54,55,56,57,58,59,60,61,62,63,64,65,66,67,68,70,71,72,73,74,75,76,77,78,79,80,81,82,83,85,86,87,88].

#	Phytochemicals or Plants	Target Regulating Mechanisms	Refs
1	Areca nut procyanidin	PEPCK and G6Pase	[54]
2	Dieckol	SOD, CAT, and GSH-px	[55]
3	Pterosin A	PEPCK	[56]
4	*Saffron*	MAPK	[57]
5	Berberine	PEPCK and G6Pase, FAS, FoxO1, SREBP1, ChREBP	[58,59,60]
6	Aloe	Adiponectin and leptin	[61]
7	*Larix laricina K. Koch*	GLUT4	[62,63]
8	Resveratrol and Piceatannol	PEPCK and G6Pase	[64,65,66]
9	Baicalein	IRS1/PI3K/Akt, SREBP1c	[67]
10	*Momordica charantia*	iNOS, NFκB, IL-1β	[68]
11	Tangeretin	Adipocytokines	[70]
12	Octaphlorethol A	Glut4 translocation, Akt	[71]
13	Chlorogenic acid	Glut4 translocation, CAMKKβ	[72,73]
14	Ginsenoside Rg1	Glut4 expression	[74]
15	Osthole	AMP: ATP ratio	[75]
16	Arctigenin	Glucose uptake	[76,77]
17	*Malva verticillata*	Glucose uptake	[78,79]
18	Karanjin	Glut4 translocation	[80]
19	*ReishiMax*	SREBP1c and C/EBPα	[81]
20	*Prunus yedoensis leaf* extract	p38 MAPK	[82,83]
21	*Artemisia sacrorum Ledeb.* (Compositae)	PGC-1α, PEPCK, G6Pase	[85]
22	*Nigella sativa* seed	Glut4 expression	[86]
23	Capsaicin	Adiponectin	[87]
24	Tang-Min-Ling	Glut4 expression	[88]

PEPCK: Phosphoenolpyruvate carboxykinase; G6Pase: Glucose 6-phosphatase; AMPK: Adenosine monophosphate-activated protein kinase; SOD: Superoxide dismutase; CAT: Catalase; GSH-px; Glutathione peroxidase; MAPK: Mitogen-activated protein kinase; FAS: Fatty acid synthase; FoxO1: Forkhead box protein O1; SREBP1: Sterol regulatory element-binding protein 1; ChREBP: Carbohydrate response element binding protein; GLUT4: Glucose transporter type 4; IRS1: Insulin receptor substrate 1; PI3K: Phosphoinositide 3-kinase; Akt: RAC-alpha serine/threonine-protein kinase; SREBP1c: sterol regulatory element-binding protein 1c; iNOS: Inducible nitric oxide synthase; NFκB: Nuclear factor kappa-light-chain-enhancer of activated B cells; IL-1β: interleukin-1beta; CAMKKβ: Ca(2+)/calmodulin-dependent protein kinase kinase beta; AMP: Adenosine monophosphate-activated protein; ATP: Adenosine triphosphate; C/EBPα: CCAAT-enhancer-binding protein alpha; PGC-1α: Peroxisome proliferator-activated receptor-gamma coactivator-1alpha.
